# The Air-Track through the Nose

**Published:** 1901-07-20

**Authors:** 


### THE AIR-TRACK THROUGH THE NOSE.

SOME curious observations have lately been made by Mr. Charles A. Parker[1] to determine the exact course which the air takes within the nose during nasal respiration. Air was impregnated with lycopodium by means of an insufflator held about eight inches from the nose during quiet and forcible inspiration, and then the disposition of the powder within the nasal cavities was noticed. The result showed that in normal noses during quiet inspiration the powder impinges on the septum about half an inch within the nostrils and a third of an inch above the floor, and is then carried upwards and backwards in a broad band into the middle and superior meatus, entirely missing the inferior passage and lower turbinate. By observing the direction taken by tobacco smoke, it was seen that during gentle expiration the air passes chiefly through the inferior meatus, that is below the upper level of the inferior turbinate. In forcible inspiration and expiration the areas passed over are somewhat wider, but the main current goes as above mentioned. Observations were also made, in the same manner, as to the direction taken by the air currents in various instances of malformation, adenoids, etc., and it was concluded that spurs and deviations of the septum and enlargements of the inferior turbinates only interfere with inspiration when they are in the way of the entrance of air into the middle meatus. Polypi or enlargements of the middle turbinates on the contrary considerably interfere with easy respiration. Hypertrophies and growths springing from the roof of the naso-pharynx also cause considerable obstruction to respiration, being in the direct air way. It may also be concluded that as in expiration the air traverses the inferior meatus chiefly, any hypertrophies or deformities causing stenosis of this passage will render expiration uncomfortable and difficult. This use of lycopodium may be of service in deciding how far an observed deformity or irregularity is really the cause of any obstruction by which it is accompanied. The powder will be freely deposited on any irregularity which is causing obstruction, while on the other hand if a spur, for example, is left untouched by the powder it may be left alone, so far as respiration is concerned, unless it is sufficiently large to interfere with expiration, which is not often the case.

[1] Lancet, July 6.

